# Comparison of outcomes and costs of surgery *versus* sclerotherapy to treat hydrocele

**DOI:** 10.31744/einstein_journal/2021GS5920

**Published:** 2021-07-08

**Authors:** Fernando Korkes, Saulo Borborema Teles, Matheus Prado Nascimento, Samira Scalso de Almeida, Artur Martins Codeço

**Affiliations:** 1 Hospital Israelita Albert Einstein Hospital Municipal Vila Santa Catarina Dr. Gilson de Cássia Marques de Carvalho São PauloSP Brazil Hospital Municipal Vila Santa Catarina Dr. Gilson de Cássia Marques de Carvalho; Hospital Israelita Albert Einstein, São Paulo, SP, Brazil.; 2 Hospital Israelita Albert Einstein São PauloSP Brazil Hospital Israelita Albert Einstein, São Paulo, SP, Brazil.; 3 Centro Universitário FMABC Santo AndréSP Brazil Centro Universitário FMABC, Santo André, SP, Brazil.; 4 Hospital Israelita Albert Einstein Faculdade Israelita de Ciências da Saúde Albert Einstein São PauloSP Brazil Faculdade Israelita de Ciências da Saúde Albert Einstein, Hospital Israelita Albert Einstein, São Paulo, SP, Brazil.

**Keywords:** Testicular hydrocele, Sclerotherapy, Cost and cost analysis, Cost-benefit analysis

## Abstract

**Objective:**

To evaluate the outcomes and costs associated with surgery *versus* sclerotherapy as treatment of hydroceles.

**Methods:**

A total of 53 men consecutively treated for hydrocele at our organization, between December 2015 and June 2019, were retrospectively analyzed (39 with Jaboulay technique and 14 with sclerotherapy). All charts were reviewed, assessing clinical data, ultrasound findings, surgical data, and post-procedure outcomes. The hospital finance department calculated the cost of outpatient evaluation, complementary tests, supplies, drugs, and professionals’ costs throughout all procedures.

**Results:**

The median age for both groups was similar (58 and 65 years old). Comorbidities were less frequent in the Surgery Group (20; 51%) than in the Sclerotherapy Group (14; 100%, p<0.05). The median length of hospital stay was 34.5±16.3 hours for the Surgery Group and 4 hours for the Sclerotherapy Group. The mean follow-up period was similar for both groups (85.4±114.8 days after surgery, and 60.9±80.1 days after sclerotherapy, p=0.467). No significant complications occurred in any patient. Success rates were 94.8% after surgery and 92.8% after sclerotherapy. The mean cost per patient was US$2,558.69 in the Surgery Group (Hydrocelectomy Group) and US$463.58 in the Sclerotherapy Group (p<0.0001). Costs directly related to in-hospital treatment procedures were significantly higher for surgery *versus* sclerotherapy (US$2,219.82±US$1,629.06 *versus* US$130.64±US$249.60; p<0.0001).

**Conclusion:**

Sclerotherapy is an excellent treatment option for idiopathic hydrocele as compared to traditional Jaboulay. It has a high success rate, low complication rates, fast discharge and patients return quicker to activities of daily living.

## INTRODUCTION

The most common form of hydrocele in adults is primary or idiopathic and affects about 1% of adult men.^(^^1^^-^^3^^)^ It is caused by an increase in fluid volume between the parietal and visceral layers of tunica vaginalis. It results from inadequate absorption of the fluid by the tunica vaginalis through lymphatic vessels.^(^^2^^)^ Most hydroceles do not require surgical treatment,^(^^4^^)^ but when they are large enough to cause bothersome symptoms, surgery has high success rates.^(^^5^^,^^6^^)^

Several surgical techniques have been described for the treatment of this condition. Sclerotherapy has also been widely performed with several agents. These distinctive approaches have varying results. Surgical treatment has high success rates but a higher number of complications, such as prolonged pain, hematoma, infection, and injury to scrotal contents.^(^^2^^,^^3^^)^ Sclerotherapy fuses the visceral and parietal layers of the tunica vaginalis, obliterating the potential space for recurrence of the hydrocele.^(^^4^^,^^7^^,^^8^^)^ It has gained broad acceptance because of its less invasive nature, low morbidity, and a faster recovery time.^(^^1^^,^^3^^,^^9^^)^ Costs are expected to be lower in the sclerotherapy method, but only a few small studies have addressed this issue in the current medical literature.^(^^4^^,^^10^^)^

## OBJECTIVE

To evaluate the outcomes and the costs associated with surgery *versus* sclerotherapy as treatment of hydrocele.

## METHODS

Study conducted at the *Hospital Municipal Vila Santa Catarina Dr. Gilson de Cássia Marques de Carvalho,* from April to May 2020.

All men treated for hydrocele between December 2015 and June 2019 at our organization were retrospectively analyzed. Patients who underwent surgical treatment through the Jaboulay technique (n=39) and sclerotherapy (n=14) were further evaluated. We excluded from the present study patients who underwent simultaneous procedures along with hydrocele treatment; treated through other surgical techniques and who were lost to follow-up.

The Jaboulay technique was performed as follows: through a median scrotal incision, the hydrocele was aspirated, the vaginal tunic excess was removed and followed by eversion over the spermatic cord. All patients were hospitalized, and spinal anesthesia was usually applied. General anesthesia was performed upon anesthesiologists’ discretion. All patients received 2g of prophylactic cephazolin preoperatively.

Sclerotherapy was routinely performed under local anesthesia, on an outpatient basis, and with ultrasound control. Patients were positioned in supine position; scrotal ultrasound was performed to evaluate the testicle and hydrocele, in addition to determining the best drainage spot. A sterile technique was used throughout the procedure. Scrotal skin anesthesia was performed with 2% lidocaine. A 16-gauge needle was inserted through ultrasound guidance, and the hydrocele was aspirated. After all, the fluid was removed, 20mL of 2% lidocaine was injected and left for 2 minutes. The lidocaine was then removed, and sclerotherapy agent (sterile alcohol 100%) was inserted according to our protocol: 10% of the removed volume up to 50mL. The needle was removed, and local compression assured for 2 minutes. Patients were observed for 1 hour and discharged, if uneventful.

All charts were reviewed, assessing clinical data, ultrasound results, surgical data, and post-procedure outcomes. The hospital finance department calculated the cost of the outpatient evaluation, complementary tests, supplies, drugs, and professionals’ costs throughout all procedures.

Recurrence was defined as any visible or palpable fluid collection that appeared and persisted after three months. For comparison of effectiveness among the techniques, up to two sclerosis procedures defined the technique successful.^(^^4^^)^ Brazilian Reais to US dollars currency rate used in the present manuscript was 5:1.

Statistical analysis was performed using (SPSS), version 20.0 (SPSS for Mac OS X, SPSS, Inc., Chicago, Illinois, USA). Groups were compared with Pearson's χ^2^ or Fisher's tests. The Student *t* test was used for continuous variables with normal distribution, and the Mann-Whitney U test for non-normal distribution variables. analysis of variance (Anova) was performed for multiple comparisons. Statistical significance was determined at p<0.05.

The Research Ethics Committee approved the present study (CAAE: 24236619.0.0000.0071, protocol 3.790.379).

## RESULTS

Demographic data are presented in table 1 . The median ages for both groups were similar (surgery 58.3±14.2 years *versus* sclerotherapy 65.3±7.9 years; p=0.1014). The body mass index (BMI; kg/m²) was 28±3.9 and 27.1±5.2 for surgery and sclerotherapy, respectively (p=0.59). Comorbidities were less common in the Surgery Group (20; 51%) than in the Sclerotherapy Group (14; 100%; p<0.05). The time between the onset of hydrocele and the first outpatient evaluation was 31.9±29.2 months for surgery *versus* 40.7±31.0 months for sclerotherapy (p=0.363). The volume of hydrocele measured through ultrasound was similar in both groups (264.0±232.7mm for Surgery and 325.1±297.8mm for Sclerotherapy; p=0.567).

**Table 1:** Demographics data

Variants	Surgery Group (n=39)	Sclerotherapy Group (n=14)	p value
Age, years	58.3±14.2	65.3±7,9	0.101
Weight, kg	81.6±12.7	82.9±15.4	0.824
Height, m	1.71±0.05	1.72±0.05	0.442
BMI, kg/m²	28±3.9	27.1±5.2	0.590
Comorbidities	20 (51)	14 (100)	0.0007
Onset of symptoms, months	31.9±29.2	40.7±31.0	0.363
Previous surgery	9 (23)	2 (14)	0.496
Volume of hydrocele in ultrasound, mL	264.0±232.7	325.1±297.8	0.567

Results expressed as mean ± standard deviation or n (%).

BMI: body mass index.

The results of perioperative data are shown in table 2 . The time between the first outpatient evaluation and the procedure was 109.8±129.5 days for surgery and 112.6±93.8 days for sclerotherapy, with no significant difference (p=0.942). The median volume aspirated was 483.2±365.9mL for surgery and 366.1±212.7mL for sclerotherapy (p=0.309). Thirty-four (87%) patients who underwent surgery received spinal anesthesia, and three (7%) had general anesthesia. In the Sclerotherapy Group, all patients were treated under local anesthesia. A drain was placed at the surgeon's discretion after each surgery and maintained for 24 hours. Drains were placed in 20 men (51%). The median length of hospital stay was 34.5±16.3 hours for the Surgery Group and 4 hours for the Sclerotherapy Group. The mean follow-up period was similar for both groups (85.4±114.8 days after surgery and 60.9±80.1 days after sclerotherapy; p=0.467). No significant complications occurred in any patient. Minor complications occurred in 18 (46%) patients after surgery, and none was found in the Sclerotherapy Group.

**Table 2:** Results of perioperative data

Variants		Surgery Group (n=39)	Sclerotherapy Group (n=14)	p value
Time to procedure, days		109.8±129.5	112.6±93.8	0.942
Volume aspirated, mL		483.2±365.9	366.1±212.7	0.309
Aspect				
	Clear	33 (84)	11 (78)	
	Turbid	1 (2)	3 (21)	
Anesthesia				
	Local	0	14 (100)	
	Spinal	34 (87)	0	
	General	3 (7)	0	
Length of hospital stay, hours		34.5±16.3	4.0±0.5	
Minor complications		18 (46)	0	0,001
Follow-up, days		85.4±114.8	60.9±80.1	0.467
First recurrence		2 (5)	7 (50)	0,003
Volume aspirated, mL		425.0±106.1	157.0±154.2	0.0794
Aspect				
	Clear	2 (100)	3 (42)	
	Turbid		2 (28)	
Second recurrence		1 (20)	1 (14)	
Volume aspirated, mL		40	139	
Aspect turbid		1 (100)	1 (100)	

Results expressed as mean ± standard deviation or n (%).

Success rates were 94.8% after surgery and 92.8% after sclerotherapy. Hydrocele recurred in two men (5%) who underwent surgery. For these patients, we performed aspiration and sclerotherapy. For seven men (50%) in the Sclerotherapy Group, a second procedure was required, and for one of these men, a third procedure was performed to obtain success ( Table 2 ).

The mean cost per patient was US$2,558.69 in the Surgery Group and US$463.58 in the Sclerotherapy Group (p<0.0001). Outpatient costs were similar in the groups (US$338.87±US$389.21 *versus* US$332.94±US$191.98; p=0.957). Cost directly related to in-hospital treatment procedures were significantly higher for surgery *versus* sclerotherapy (US$2,219.82±US$1,629.06 *versus* US$130.64±US$249.60; p<0.0001) ( Table 3 ).

**Table 3:** Costs (US$)

Variants	Surgery Group	Sclerotherapy Group	p value
Consultations	338.87±389.21	332.94±191.98	0.957
Hospital procedure	2,219.82±1,629.06	130.64±249.60	<0.0001
Total	2,558.69±1,891.94	463.58±248.40	<0.0001

Results expressed as mean±standard deviation.

## DISCUSSION

There are different treatment options for patients with hydrocele: observation, aspiration and sclerotherapy, and surgery. Hydrocelectomy is considered the gold standard.^(^^1^^)^ However, it needs to be performed in the operating room, often with spinal or general anesthesia, increasing the cost of care compared to sclerotherapy.^(^^9^^,^^10^^)^ Since it occurs mainly in young adults, it leads to loss of working days that can be seven times longer in hydrocelectomy compared to sclerotherapy.^(^^10^^)^ Our study aimed to evaluate the costs associated with these modalities applied to treat hydrocele in adults.

This study has some significant findings. First, we have observed a much higher cost associated with the surgical procedures when compared to sclerotherapy (US$2,558.69 *versus* US$463.58; p<0.0001). It represents an economy of 81% or US$2,095.11 for each patient treated by sclerotherapy. Surgery was 5.5-fold more expensive than sclerotherapy.

Even though costs associated with hydrocele treatment are not exceedingly high, it is not an uncommon disease. After several procedures performed there are significant savings, which can be even more relevant in a developing country such as ours, where resources are scarce. Other authors have shown that hydrocelectomy can bear a nine-fold higher cost than sclerotherapy.^(^^4^^)^

Additionally, sclerotherapy was performed as an outpatient procedure. It brings the advantage over inpatient surgery not only regarding the costs but also convenience for patients. Hydrocelectomy can also be performed as an outpatient procedure, even though associated with higher complication rates.^(^^11^^,^^12^^)^

Our hospital receives patients from the public health system. Most of our patients live far from our facilities, and therefore it is our choice not to perform hydrocelectomy as an outpatient procedure. For sclerotherapy, since risks are low, we adopted the outpatient routine.

Second, success rates were high for all procedures. Sclerotherapy has achieved a 92.8% success after two procedures and 100% success after three procedures. Hydrocelectomy had only two failures in our series, both successfully treated through sclerotherapy. The definition of success in the literature varies widely but ranges from 44% to 100%.^(^^4^^,^^10^^)^ Higher success rates of sclerotherapy can be achieved by increasing the number of treatments offered to patients before surgical options were explored.^(^^4^^)^ Sclerotherapy is, therefore, a good option for the treatment of hydrocele, mainly in elderly patients or those unfit for surgery, as also observed by other authors.^(^^5^^)^ Other advantages are that the patient does not need fasting, can maintain usual medications, and does not require spine or general anesthesia and their inherent risks.

Complications were more common after surgical procedures, and no events were reported in the Sclerotherapy Group. Even though complications have been reported after sclerotherapy, they are uncommon.^(^^4^^)^ A concern in young patients is spermatogenesis. Shan et al., have addressed this issue, and no significant impairment in spermatogenesis or fertility occurred after sclerotherapy, assuring the safety of this procedure even in young men.^(^^8^^)^

Our study has some limitations. It was retrospective, and a relatively small number of patients were evaluated. However, we do not require a massive number of patients to allow further conclusions for cost analysis. Additionally, only a few papers have analyzed the cost associated with hydrocele treatment, and none was carried out in Brazil.

## CONCLUSION

Sclerotherapy is an excellent treatment option for idiopathic hydrocele comparing to traditional Jaboulay. It has a high success rate, low complication rates, fast discharge and patients return quicker to activities of daily living. No drain and major anesthesia are required. The recurrence seems to be similar after both procedures, but costs are significantly lower after sclerotherapy.
