# Supplementary material for: Comparison of outcomes and costs of surgery versus sclerotherapy to treat hydrocele
Source: Einstein (Sao Paulo). 2021 Jul 8;19:eGS5920. doi: 10.31744/einstein_journal/2021GS5920 (PMC8277233; doi:10.31744/einstein_journal/2021GS5920)
Supplement: Translation

# Translation: Comparação entre resultados e custos de cirurgia e de escleroterapia para tratamento de hidrocele

**Authors:** Fernando Korkes, Saulo Borborema Teles, Matheus Prado Nascimento, Samira Scalso de Almeida, Artur Martins Codeço

**Affiliations:** 1 São PauloSP Brasil Hospital Municipal Vila Santa Catarina Dr. Gilson de Cássia Marques de Carvalho; Hospital Israelita Albert Einstein, São Paulo, SP, Brasil.; 2 São PauloSP Brasil Hospital Israelita Albert Einstein, São Paulo, SP, Brasil.; 3 Santo AndréSP Brasil Centro Universitário FMABC, Santo André, SP, Brasil.; 8 São PauloSP Brasil Faculdade Israelita de Ciências da Saúde Albert Einstein, Hospital Israelita Albert Einstein, São Paulo, SP, Brasil.

**Keywords:** Hidrocele testicular, Escleroterapia, Custo e análise de custo, Análise custo-benefício

## Abstract

**Objetivo:**

Avaliar resultados e custos associados à cirurgia e à escleroterapia como tratamentos das hidroceles.

**Métodos:**

Foram tratados consecutivamente para hidrocele em nossa instituição 53 homens, entre dezembro de 2015 e junho de 2019, os quais foram analisados retrospectivamente (39 pela técnica de Jaboulay e 14 por escleroterapia). Todos os prontuários foram revisados, avaliando dados clínicos, achados de ultrassom, dados cirúrgicos e desfechos pós-procedimento. O departamento financeiro do hospital calculou o custo da avaliação ambulatorial, dos exames complementares, dos insumos, dos medicamentos e dos profissionais em todos os procedimentos.

**Resultados:**

A idade mediana foi semelhante nos dois grupos (58 *e* 65 anos). Comorbidades foram menos frequentes no Grupo Cirurgia (20; 51%) do que no Grupo Escleroterapia (14; 100%; p<0,05). O tempo mediano de internação hospitalar foi 34,5±16,3 horas para o Grupo Cirurgia e 4 horas para Grupo Escleroterapia. O período médio de seguimento foi semelhante nos dois grupos (85,4±114,8 dias após a cirurgia e 60,9±80,1 dias após escleroterapia; p=0,467). Nenhuma complicação significativa ocorreu nos pacientes. As taxas de sucesso foram de 94,8% após a cirurgia e 92,8% após a escleroterapia. O custo médio por paciente foi de US$2,558.69 para Grupo Cirurgia e US$463.58 para Grupo Escleroterapia (p<0,0001). Os custos relacionados aos procedimentos de tratamento hospitalar foram significativamente maiores para cirurgia em relação à escleroterapia (US$2,219.82±US$1,629.06 *versus* US$130.64±US$249.60; p<0,0001).

**Conclusão:**

A escleroterapia é uma excelente opção de tratamento para hidrocele idiopática em comparação com a tradicional Jaboulay. Apresenta alta taxa de sucesso, baixas taxas de complicações e alta rápida, além de os pacientes retornarem mais rapidamente às atividades diárias.

## INTRODUÇÃO

A forma mais comum de hidrocele em adultos é a primária ou idiopática e afeta cerca de 1% dos homens adultos.^(^^1^^-^^3^^)^É causada por um aumento no volume de fluido entre as camadas parietal e visceral da túnica vaginal. Resulta da absorção inadequada do fluido pela túnica vaginal através dos vasos linfáticos.^(^^2^^)^A maioria das hidroceles não requer tratamento cirúrgico,^(^^4^^)^mas quando elas são grandes o suficiente para causar sintomas incômodos, a cirurgia tem altas taxas de sucesso.^(^^5^^,^^6^^)^

Diversas técnicas cirúrgicas foram descritas para o tratamento dessa condição. A escleroterapia também tem sido amplamente realizada com vários agentes. Essas diferentes abordagens têm resultados variados. O tratamento cirúrgico tem altas taxas de sucesso, mas um maior número de complicações, como dor prolongada, hematoma, infecção e lesão do conteúdo escrotal.^(^^2^^,^^3^^)^A escleroterapia funde as camadas visceral e parietal da túnica vaginal, obliterando o espaço potencial para a recorrência da hidrocele.^(^^4^^,^^7^^,^^8^^)^Ganhou ampla aceitação por sua natureza menos invasiva, baixa morbidade e um tempo de recuperação mais rápido.^(^^1^^,^^3^^,^^9^^)^Espera-se que os custos sejam menores usando-se o método da escleroterapia, mas apenas alguns pequenos estudos abordaram essa questão na literatura médica atual.^(^^4^^,^^10^^)^

## OBJETIVO

Avaliar os resultados e os custos associados à cirurgia em comparação aos da escleroterapia como tratamento da hidrocele.

## MÉTODOS

Estudo realizado no Hospital Municipal Vila Santa Catarina Dr. Gilson de Cássia Marques de Carvalho, de abril a maio de 2020.

Todos os homens tratados para hidrocele entre dezembro de 2015 e junho de 2019 em nossa instituição foram analisados retrospectivamente. Os pacientes submetidos a tratamento cirúrgico pela técnica de Jaboulay (n=39) e por escleroterapia (n=14) foram avaliados minuciosamente. Excluímos do presente estudo os pacientes submetidos a procedimentos simultâneos para tratamento da hidrocele, tratados por meio de outras técnicas cirúrgicas e que deixaram de ser acompanhados.

A técnica de Jaboulay foi realizada da seguinte forma: por meio de incisão escrotal mediana, aspirou-se a hidrocele e retirou-se o excesso da túnica vaginal, seguindo-se de eversão sobre o cordão espermático. Todos os pacientes foram hospitalizados, sendo-lhes geralmente aplicada raquianestesia. A anestesia geral foi realizada a critério do anestesiologista. Todos os pacientes rece­beram 2g de cefazolina profilática no pré-operatório.

A escleroterapia foi realizada de rotina sob anestesia local, em regime ambulatorial e com controle ultrassonográfico. Os pacientes foram colocados em decúbito dorsal, sendo realizada ultrassonografia escrotal para avaliação do testículo e da hidrocele, além de determinar o melhor local de drenagem. Uma técnica estéril foi usada durante todo o procedimento. A anestesia da pele escrotal foi realizada com lidocaína a 2%. Uma agulha calibre 16 foi inserida por meio de ultrassom, e a hidrocele foi aspirada. Por fim, o líquido foi removido, e foram injetados 20mL de lidocaína a 2% e deixados por 2 minutos. A lidocaína foi, então, removida, e o agente de escleroterapia (álcool estéril 100%) foi inserido de acordo com nosso protocolo: 10% do volume removido até 50mL. A agulha foi retirada, mantendo-se compressão local por 2 minutos. Os pacientes foram observados por 1 hora e receberam alta caso não tivessem intercorrências.

Todos os prontuários foram examinados, sendo avaliados dados clínicos e cirúrgicos, resultados de exames ultrassonográficos e desfechos pós-procedimento. O departamento financeiro do hospital calculou o custo da avaliação ambulatorial, dos exames complementares, dos insumos, dos medicamentos e dos profissionais em todos os procedimentos.

A recorrência foi definida como qualquer coleção de fluido visível ou palpável que tenha aparecido e persistido após 3 meses. Para comparação da efetividade entre as técnicas, até dois procedimentos de esclerose definiram o sucesso da técnica.^(^^4^^)^A taxa de câmbio entre Reais e dólares americanos utilizada foi de 5:1.

A análise estatística foi realizada usando o software (SPSS), versão 20.0 (SPSS para Mac OS X, SPSS, Inc., Chicago, Illinois, USA). Os grupos foram comparados com o teste do χ^2^de Pearson ou o teste de Fisher. O teste *t* de Student foi empregado para variáveis contínuas com distribuição normal, e o teste U de Mann-Whitney para variáveis de distribuição não normal. Foi realizada análise de va­riância (Anova) para comparações múltiplas. A significância estatística foi determinada em p<0,05.

O Conselho de Ética em Pesquisa aprovou o presente estudo (CAAE: 24236619.0.0000.0071, protocolo 3.790.379).

## RESULTADOS

Os dados demográficos são apresentados na tabela 1 . As idades medianas para ambos os grupos foram semelhantes (cirurgia 58,3±14,2 anos *versus* escleroterapia 65,3±7,9 anos; p=0,1014). O índice de massa corporal (IMC; kg/m²) foi de 28±3,9 e 27,1±5,2 para cirurgia e escleroterapia, respectivamente (p=0,59). As comorbidades foram menos comuns no Grupo Cirurgia (20; 51%) do que no Grupo Escleroterapia (14; 100%; p<0,05). O tempo entre o início da hidrocele e a primeira avaliação ambulatorial foi de 31,9±29,2 meses para a cirurgia *versus* 40,7±31,0 meses para escleroterapia (p=0,363). O volume de hidrocele medido por ultrassom foi semelhante nos dois grupos (264,0±232,7mm para cirurgia e 325,1±297,8mm para escleroterapia; p=0,567).

**Tabela 1:** Dados demográficos

Variantes	Grupo Cirurgia (n=39)	Grupo Escleroterapia (n=14)	Valor de p
Idade, anos	58,3±14,2	65,3±7,9	0,101
Peso, kg	81,6±12,7	82,9±15,4	0,824
Altura, m	1,71±0,05	1,72±0,05	0,442
IMC, kg/m^2^	28±3,9	27,1±5,2	0,590
Comorbidades	20 (51)	14 (100)	0,0007
Início dos sintomas, meses	31,9±29,2	40,7±31,0	0,363
Cirurgia prévia	9 (23)	2 (14)	0,496
Volume da hidrocele no ultrassom, mL	264,0±232,7	325,1±297,8	0,567

Resultados expressos por média±desvio padrão ou n (%).

IMC: índice de massa corpórea.

Os resultados dos dados perioperatórios são apresentados na tabela 2 . O tempo entre a primeira avaliação ambulatorial e o procedimento foi de 109,8±129,5 dias para a cirurgia e 112,6±93,8 dias para escleroterapia, sem diferença significativa (p=0,942). O volume mediano aspirado foi de 483,2±365,9mL para cirurgia e 366,1±212,7mL para escleroterapia (p=0,309). Trinta e quatro (87%) pacientes operados receberam raquianestesia, e três (7%) receberam anestesia geral. No Grupo Escleroterapia, todos os pacientes foram tratados sob anestesia local. Um dreno foi colocado a critério do cirurgião após cada cirurgia e mantido por 24 horas. Foram colocados drenos em 20 homens (51%). A mediana do tempo de internação hospitalar foi 34,5±16,3 horas para o Grupo Cirurgia e 4 horas para o Grupo Escleroterapia. O período médio de acompanhamento foi semelhante para os dois grupos (85,4±114,8 dias após a cirurgia e 60,9±80,1 dias após a escleroterapia; p=0,467). Não ocorreu complicação significativa em nenhum paciente. Ocorreram complicações menores em 18 (46%) pacientes após a cirurgia, e nenhuma foi encontrada no Grupo Escleroterapia.

**Tabela 2:** Resultados dos dados perioperatórios

Variantes		Grupo Cirurgia (n=39)	Grupo Escleroterapia (n=14)	Valor de p
Tempo até o procedimento, dias		109,8±129,5	112,6±93,8	0,942
Volume aspirado, mL		483,2±365,9	366,1±212,7	0,309
Aspecto				
	Claro	33 (84)	11 (78)	
	Turvo	1 (2)	3 (21)	
Anestesia				
	Local	0	14 (100)	
	Raqui	34 (87)	0	
	Geral	3 (7)	0	
Tempo de hospitalização, horas		34,5±16,3	4,0±0,5	
Complicações menores		18 (46)	0	0.001
Acompanhamento, dias		85,4±114,8	60,9±80,1	0,467
Primeira recorrência		2 (5)	7 (50)	0,003
Volume aspirado, mL		425,0±106,1	157,0±154,2	0,0794
Aspecto				
	Claro	2 (100)	3 (42)	
	Turvo	0	2 (28)	
Segunda recorrência		1 (20)	1 (14)	
Volume aspirado, mL		40	139	
Aspecto turvo		1 (100)	1 (100)	

Resultados expressos por média±desvio padrão ou n (%).

As taxas de sucesso foram de 94,8% após cirurgia e 92,8% após escleroterapia. Houve recorrência da hidrocele em dois homens (5%) operados. Para esses pacientes, realizamos aspiração e escleroterapia. Para sete homens (50%) do Grupo Escleroterapia, foi necessário um segundo procedimento e, para um desses homens, um terceiro procedimento foi realizado para obter sucesso ( Tabela 2 ).

O custo médio por paciente foi de US$2,558.69 no Grupo Cirurgia e US$463.58 no Grupo Escleroterapia (p<0,0001). Os custos ambulatoriais foram semelhantes nos grupos (US$338.87±US$389.21 *versus* US$332.94±US$191.98; p=0,957). Os custos diretamente relacionados aos procedimentos terapêuticos durante a internação hospitalar foram significativamente maio­res para cirurgia em comparação à escle­roterapia (US$2,219.82±US$1,629.06 *versus* US$130.64±US$249.60; p<0,0001) ( Tabela 3 ).

**Tabela 3:** Custos (US$)

Variantes	Grupo Cirurgia	Grupo Escleroterapia	Valor de p
Consultas	338.87±389.21	332.94±191.98	0,957
Procedimento hospitalar	2,219.82±1,629.06	130.64±249.60	<0,0001
Total	2,558.69±1,891.94	463.58±248.40	<0,0001

Resultados expressos por média±desvio padrão.

## DISCUSSÃO

Existem diferentes opções de tratamento para pacientes com hidrocele: observação, aspiração e escleroterapia, além da cirurgia. A hidrocelectomia é considerada o padrão-ouro.^(^^1^^)^No entanto, precisa ser realizada em sala de cirurgia, muitas vezes sob anestesia raquidiana ou geral, aumentando o custo do atendimento em comparação à escleroterapia.^(^^9^^,^^10^^)^Uma vez que ocorre principalmente em adultos jovens, resulta em perda de dias de trabalho, que pode ser sete vezes maior na hidrocelectomia, em comparação com a escleroterapia.^(^^10^^)^Nosso estudo teve como objetivo avaliar os custos associados a essas modalidades aplicadas no tratamento de hidrocele em adultos.

Este estudo tem alguns achados significativos. Em primeiro lugar, observamos um custo muito maior associado aos procedimentos cirúrgicos em comparação à escleroterapia (US$2,558.69 *versus* US$463.58; p<0,0001). Isso representa uma economia de 81% ou US$2,095.11 para cada paciente tratado por escleroterapia. A cirurgia foi 5,5 vezes mais cara que a escleroterapia.

Embora os custos associados ao tratamento da hidrocele não sejam excessivamente altos, essa não é uma doença incomum. Depois de vários procedimentos rea­lizados, há uma economia significativa, que pode ser ainda mais relevante em um país em desenvolvimento como o nosso, onde os recursos são escassos. Outros autores demonstraram que a hidrocelectomia pode ter um custo nove vezes maior do que a escleroterapia.^(^^4^^)^

Além disso, a escleroterapia foi realizada em regime ambulatorial. Apresenta vantagem sobre a cirurgia realizada em pacientes internados não só no que diz respeito aos custos, mas também em termos de comodidade para os pacientes. A hidrocelectomia também pode ser realizada ambulatorialmente, embora associada a maiores taxas de complicações.^(^^11^^,^^12^^)^

Nosso hospital recebe pacientes da rede pública de saúde. A maioria de nossos pacientes mora longe de nossas instalações, e, portanto, é nossa escolha não re­a­lizar a hidrocelectomia como um procedimento ambulatorial. Para a escleroterapia, como os riscos são baixos, adotamos a rotina ambulatorial.

Em segundo lugar, as taxas de sucesso foram altas para todos os procedimentos. A escleroterapia obteve sucesso de 92,8% após dois procedimentos e de 100% de sucesso após três procedimentos. A hidrocelectomia teve apenas duas falhas em nossa série, ambas tratadas com sucesso por meio da escleroterapia. A definição de sucesso na literatura varia amplamente, indo de 44% a 100%.^(^^4^^,^^10^^)^Podem ser alcançadas maiores taxas de sucesso de escleroterapia ao aumentar o número de tratamentos oferecidos aos pacientes antes que as opções cirúrgicas sejam exploradas.^(^^4^^)^A escleroterapia é, portanto, uma boa opção para o tratamento da hidrocele, principalmente em pacientes idosos ou inaptos para a cirurgia, como também observado por outros autores.^(^^5^^)^Outras vantagens são a não necessidade de jejum, manter os medicamentos usuais e não requerer anestesia raquidiana ou geral, afastando, assim, seus riscos inerentes.

As complicações foram mais comuns após procedimentos cirúrgicos, e nenhum evento foi relatado no Grupo Escleroterapia. Embora tenham sido relatadas complicações após a escleroterapia, elas são incomuns.^(^^4^^)^Uma preocupação em pacientes jovens é a espermatogênese. Shan et al., abordaram essa questão, e nenhum prejuízo significativo na espermatogênese ou fertilidade ocorreu após a escleroterapia, garantindo a segurança desse procedimento mesmo em homens jovens.^(^^8^^)^

Nosso estudo tem algumas limitações. Ele foi retrospectivo e um número relativamente pequeno de pacientes foi avaliado. No entanto, não precisamos de um grande número de pacientes para permitir conclusões adicionais no tocante à análise de custos. Além disso, poucos estudos analisaram o custo associado ao tratamento da hidrocele, e nenhum deles foi realizado no Brasil.

## CONCLUSÃO

A escleroterapia é uma excelente opção de tratamento para hidrocele idiopática em comparação com a traditional técnica de Jaboulay. Apresenta altas taxas de sucesso, baixas taxas de complicações, alta rápida e retorno mais rápido às atividades diárias. Não requer dreno nem anestesia de grande porte. A recorrência parece ser semelhante após os dois procedimentos, mas os custos são significativamente menores após a escleroterapia.
